# *SIRT1* Gene Polymorphisms Affect the Protein Expression in Cardiovascular Diseases

**DOI:** 10.1371/journal.pone.0090428

**Published:** 2014-02-28

**Authors:** Ulkan Kilic, Ozlem Gok, Ahmet Bacaksiz, Muzeyyen Izmirli, Birsen Elibol-Can, Omer Uysal

**Affiliations:** 1 Department of Medical Biology, Faculty of Medicine, Bezmialem Vakif University, Istanbul, Turkey; 2 Department of Cardiology, Faculty of Medicine, Bezmialem Vakif University, Istanbul, Turkey; 3 Department of Medical Biology, Faculty of Medicine, Mustafa Kemal University, Hatay, Turkey; 4 Department of Biostatistics, Faculty of Medicine, Bezmialem Vakif University, Istanbul, Turkey; Sudbury Regional Hospital, Canada

## Abstract

Cardiovascular disease (CVD), the leading cause of death worldwide, is related to gene-environment interactions due to epigenetic factors. SIRT1 protein and its downstream pathways are critical for both normal homeostasis and protection from CVD-induced defects. The aim of this study was to investigate the association between *SIRT1* single nucleotide polymorphisms (SNPs) (rs7895833 A>G in the promoter region, rs7069102 C>G in intron 4 and rs2273773 C>T in exon 5 silent mutation) and SIRT1 and eNOS (endothelial nitric oxide synthase) protein expression as well as total antioxidant status (TAS), total oxidant status (TOS) and oxidative stress index (OSI) in CVD patients as compared to controls. The frequencies of mutant genotypes and alleles for rs7069102 and rs2273773 were significantly higher in patients with CVD compared to control group. The risk for CVD was increased by 2.4 times for rs7069102 and 1.9 times for rs2273773 in carriers of mutant allele compared with carriers of wild-type allele pointing the protective role of C allele for both SNPs against CVD. For rs7895833, there was no significant difference in genotype and allele distributions between groups. SIRT1 protein, TAS, TOS and OSI levels significantly increased in patients as compared to control group. In contrast, level of eNOS protein was considerably low in the CVD patients. An increase in the SIRT1 expression in the CVD patients carrying mutant genotype for rs7069102 and heterozygote genotype for all three SNPs was observed. This is the first study reporting an association between *SIRT1* gene polymorphisms and the levels of SIRT1 and eNOS expressions as well as TAS, TOS and OSI.

## Introduction

Cardiovascular disease (CVD), the leading cause of death worldwide, is related gene-environment interaction due to epigenetic factors [1] such as smoking, hyperlipidemia, hypertension, age, family history, diabetes mellitus, and obesity. The prevalence of CVD among in Turkey’s 70.5 million people (low median age of 29) is estimated as 21.7% [2]. After the determination of genetic risk factors related CVD at earlier ages, the life coaching related with modifiable risk factors may decrease the risk of CVD at older ages. Therefore, because of its high medical risks and prevalence, research of genetic backgrounds of CVD is immediately needed.

In the previous candidate gene, linkage analysis, and genome-wide association studies, more than a dozen of genes and genetic loci related with CVD were determined [3]–[5]. The sirtuin protein family is thought as one of the important target for CVD development [6]. SIRT1 protein, a member of Sirtuin or Sir2 protein (Silent Information Regulator 2) family, consists of seven members in humans *(SIRT1-7)* [7]. *SIRT1* gene, consisting of 9 exons and 8 introns, is located on chromosome 10p37.5 (PUBMED, gene). SIRT1 protein, a NAD-dependent histone deacetylase, is expressed in many tissue and organ systems such as liver, spleen, kidney, brain, heart, pancreas, endothelial tissue, skeletal muscle and white adipose tissue [8]–[11]. While it is expressed in both the cytoplasm and nucleus, it is dominantly located in the nucleus. It acts as a transcription factor and cofactor in addition to being a target for histone and non-histone proteins [7], [8].

*SIRT1*, known as a longevity gene, protects cells against oxidative stress, promotes DNA stability by binding to several substrates and deacetylating these substrates. In the cardiovascular system, SIRT1 activation exerts multiple protective effects through distinct metabolic and stress response pathways. SIRT1 expression modulates its downstream pathways by targeting many cellular proteins, such as peroxisome proliferators-activated receptor-γ (PPAR-γ) and its coactivator-1α (PGC-1α), forkhead transcriptional factors (FoxOs), AMP-activated protein kinase (AMPK), nuclear factor-κB (NF-κB), protein tyrosine phosphatase (PTP), endothelial nitric oxide synthase (eNOS), and p53. PGC-1α protects the endothelial cells from apoptosis by preventing reactive oxygen species (ROS) mediated cardiotoxicity [12], [13]. In cardiomyocytes, due to its antioxidant activity, nuclear SIRT1 increases the resistance of myoblast to oxidative stress by enhancing the MnSOD expression through p53 deacetylation [14]. Protection of cardiomyocytes from oxidative stress also regulated by overexpression of SIRT1 protein and activation of FoxO1-dependent pathway [15]. The activation of this pathway also reduces cardiac infarct volume and improves functional recovery after ischemia/reperfusion in mice [16]. SIRT1 improves endothelial function to prevent atherosclerosis by improving endothelium relaxation through up-regulating eNOS expression and production of nitric oxide [17].

Although its roles in CVD development were already reported, the underlying molecular mechanism of genetic causes of CVD requires more investigations to better elucidate safe and effective strategies for prevention and treatment. Previous studies showed that some of the *SIRT1* SNPs are associated with body mass index and obesity, glucose tolerance and diabetes, blood pressure, cholesterol metabolism and coronary artery calcification which may provide cardiovascular phenotype [7], [18]–[21]. Because it may be thought to affect the CVD phenotype, we analyzed three candidate polymorphism from promoter, intron and exon regions of SIRT1 gene. The aim of this study was to investigate the association between *SIRT1* single nucleotide polymorphisms (rs7895833 A>G in the promoter region, rs7069102 C>G in intron 4 and rs2273773 C>T in exon 5 silent mutation) and levels of SIRT1 and eNOS expression, as well as total antioxidant status (TAS), total oxidant status (TOS) and oxidative stress index (OSI) in Turkish CVD patients as compared to controls.

## Experimental Methods

### Study Groups

Corresponding to power analysis (80% power with 95% confidence interval), the study groups consisted of 278 patients who underwent coronary angiography (61 female and 217 male and mean age: 58.99±10.56) and 135 control subjects (15 female and 120 male and mean age: 48.52±5.87). Patients enrolled in this study were selected from people who underwent elective diagnostic conventional coronary angiography for suspected CAD (Coronary Artery Disease) at our institution (Bezmialem Vakif University Hospital, Department of Cardiology). Patients whose coronary angiogram showed coronary artery stenosis were included in the current study. The patients who had histories of recent myocardial infarction (MI), unstable angina pectoris (UAP), heart failure (systolic and/or diastolic heart failure was excluded with the use of transthoracic echocardiography, left ventricular ejection fraction <40% was accepted as systolic heart failure), moderate-to-severe heart valve disease, malignancies, major trauma or surgery in the previous six months, renal and/or hepatic insufficiency, acute or chronic infectious disease, any kind of immune-mediated disease were excluded. The randomly selected healthy controls were also recruited from people who came to Bezmialem Vakif University Hospital for routine examination and they and their families had no prior history of cardiovascular diseases.

This study was approved by the Ethical Committee of Bezmialem Vakif University, Faculty of Medicine. All of participants, after giving written informed consent, completed a structured questionnaire in order to collect demographic data The study was conducted in accordance with the ethical principles described by the Declaration of Helsinki.

### Biochemical and Demographic Analysis

Blood samples were obtained after 12 h of fasting by taking into plain tubes (Vacuette, Greiner Labor technic, Germany). The samples in tubes were centrifuged for 5 min at 4.500 rpm at +4°C, followed by the removal of serum and plasma and then were stored at −20°C. The following biochemical parameters were determined in both the control and experimental groups by standard laboratory methods in the Bezmialem Vakif University Hospital: fasting glucose, total cholesterol, triglyceride, high-density lipoprotein (HDL) cholesterol and low-density lipoprotein (LDL) cholesterol. Body mass index (BMI) was calculated by dividing weight by height square (kg/m^2^) and categorized according to World Health Organization recommendations.

### Determination of SIRT1 and eNOS Protein Levels

Plasma samples of subjects were analyzed for the levels of SIRT1 and eNOS proteins by using enzyme-linked immunosorbent assay (ELISA) kits from USCN Life Science (Catalog no: E94912Hu for SIRT1 and E908868Hu for eNOS, Wuhan/CHINA).

Standards and samples were incubated with antibody-coated 96-well plates. Then, enzyme-linked antibodies for the proteins were added. The intensity of the color was measured in a microplate reader (Chromate Manager 4300, Palm City/USA) at 450 nm.

### Measurement of Total Antioxidant and Oxidant Status

The total antioxidant status (TAS) and the total oxidant status (TOS) of serum was determined using an automated measurement method [22], [23] by an automated analyzer (Chromate Manager 4300, Palm City/USA). Briefly, in the measurement of TAS, potent free-radical reactions were initiated with the production of a hydroxyl radical via the Fenton reaction, and the rates of reactions were monitored by following the absorbance of colored dianisidyl radicals. Using this method, the antioxidative effect of the sample against potent free-radical reactions, which were initiated by the synthesized hydroxyl radical, was measured (Rel Assay Diagnostics, Gaziantep/TURKEY). Data were expressed as mmol equiv/L Trolox.

In the measurement of TOS, oxidants present in the sample oxidize ferrous iono-dianisidine complex to ferric ion. The oxidation reaction is enhanced by glycerol molecules, which are abundantly present in the reaction medium. Ferric ion reacts with xylenol orange in an acidic medium to produce a colored complex. The intensity of the color is related to the total amount of oxidant molecules in the sample. The assay is calibrated with hydrogen peroxide and results are expressed in terms of micromolar hydrogen peroxide (H_2_O_2_) equivalents per liter (µmol H_2_O_2_ equiv/L) (Rel Assay Diagnostics, Gaziantep/TURKEY).

### Calculation of the Oxidative Stress Index

In our study, oxidative stress index (OSI) was formulated as [24]:
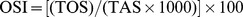



### DNA Isolation

Blood samples of all subjects were taken to the tubes containing EDTA and the genomic DNA was isolated from peripheral blood leukocytes with DNA isolation kit (Invitrogen, Carlsbad, USA). All purified DNA samples were stored at +4°C until PCR application were performed [25], [26].

### Determination of SIRT1 Gene Polymorphisms

*SIRT1* rs7895833 A>G in the promoter region, rs7069102 C>G in intron 4 and rs2273773 C>T in exon 5 gene polymorphisms [7] were analyzed using PCR-CTPP assay as described previously with minor modifications [27], [28].

**Figure 1:**
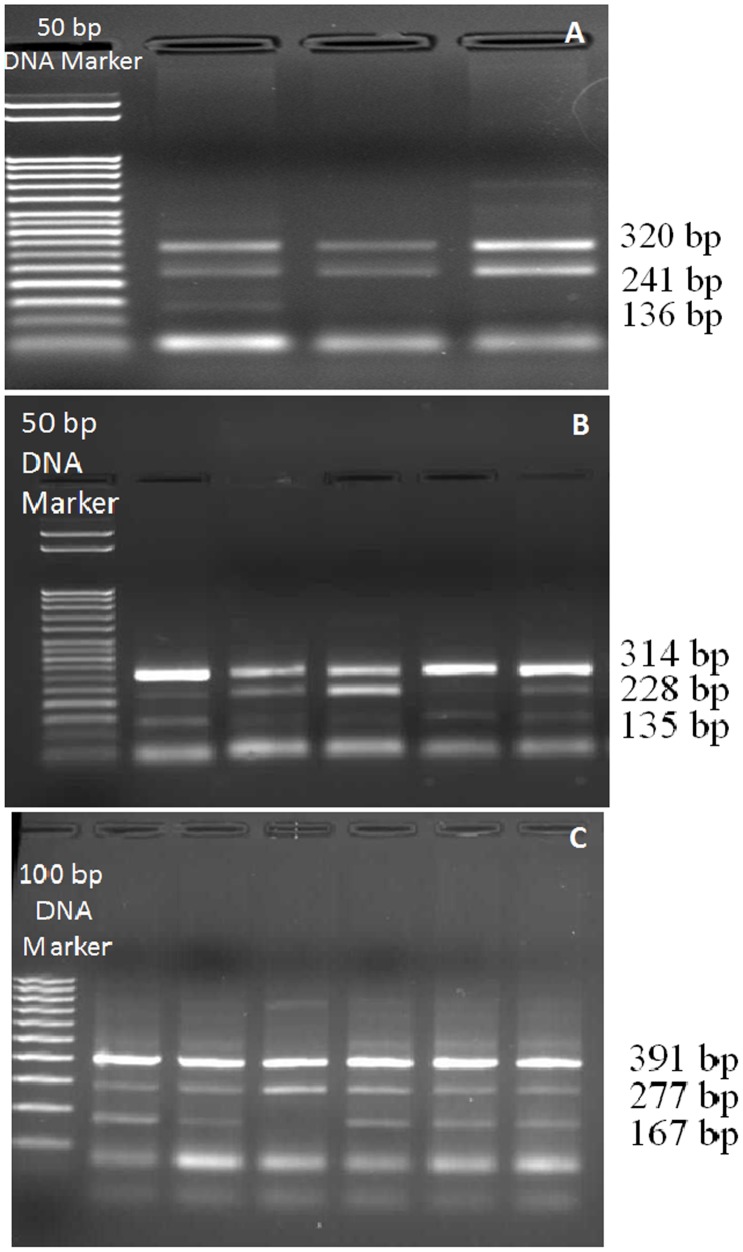
Representative gel images showing of each *SIRT1* gene polymorphisms; A. rs7895833 containing a 50 bp DNA ladder in the first lane. B. rs2273773 containing a 50

Three different segments of the *SIRT1* gene encompassing rs7895833 A>G, rs7069102 C>G, rs2273773 C>T polymorphic sites were amplified by PCR using the primers as described previously [7]. Briefly, 25 µl total PCR mixtures containing 100–200 ng DNA, 10.0 pmol of each primers, 1.0 mM dNTP (deoxynucleotide triphosphates), 25 mM MgCl_2_ and 2.5U Taq DNA polymerase in the supplied reaction buffer (Taq Buffer with (NH_4_)_2_SO_4_) were prepared. PCR was performed with the primers as shown in Table 1, with the initial denature at 95°C for 10 min.; 30 cycles of 95°C for 1 min., 64°C for **rs7895833 A>G polymorphism**, 62°C for **rs7069102 C>G polymorphism,** 63°C for **rs2273773 C>T polymorphism** for 1 min.**,** and 72°C for 1 min. and additionally the final step at 72°C for 5 min. PCR products were visualized on a 2% agarose gel with ethidium bromide staining and genotyped. Three genotypes for each polymorphism were defined by 3 distinct banding patterns (Figure 1); for **rs7895833 A>G polymorphism:** 320, 241 bp for AA genotype; 320, 241 and 136 bp for AG genotype; and 320, 136 bp for GG genotype; for **rs7069102 C>G polymorphism:** 391, 277 bp for CC genotype; 391, 277, 167 bp for CG genotype; and 391, 167 bp for GG genotype; for **rs2273773 C>T polymorphism:** 314, 228 bp for CC genotype; 314, 228, 135 bp for CT genotype; and 314, 135 bp for TT genotype [7].

**Table 1:** Primer sequences in *SIRT1* gene polymorphisms.

Polymorphism	Primer Sequences
rs7895833 A>G	Forward primer 1: CCCAGGGTTCAACAAATCTATGTTG
	Forward primer 2: GGTGGTAAAAGGCCTACAGGAAA
	Reverse primer 1: GCTTCCTAATCTCCATTACGTTGAC
	Reverse primer 2: CCTCCCAGTCAACGACTTTATC
rs7069102 C>G	Forward primer 1: GTAGCAGGAACTACAGGCCTG
	Forward primer 2: GAGAAGAAAGAAAGGCATAATCTCTGC
	Reverse primer 1: CTATCTGCAGAAATAATGGCTTTTCTC
	Reverse primer 2: GATCGAGACCATCCTGGCTAAG
rs2273773 C>T	Forward primer 1: GTGTGTCGCATCCATCTAGATAC
	Forward primer 2: CTCTCTGTCACAAATTCATAGCCT
	Reverse primer 1: GTAGTTTTCCTTCCTTATCTGACAG
	Reverse primer 2: CTGAAGTTTACTAACCATGACACTG

### Statistical Evaluation

Statistical analyses of differences in the distribution of the genotypes or alleles in *SIRT1* gene between patients and control groups were tested by Chi-Square (χ2) test using a standard software package (SPSS 18 for Windows; SPSS Inc., Chicago, IL, USA). Clinical characteristics and protein levels were compared by Student’s t-test and One way ANOVA using the same software. Relative risk at 95% confidence intervals (CI) was calculated as the odds ratio (OR). Pearson’s correlation test was also applied to determine the relation between the SIRT1 expression level and other parameters. A p value of less than 0.05 was regarded as being statistically significant.

## Results

### Biochemical and Demographic Analysis

Demographic and clinical characteristics of subjects are summarized in Table 2. According to the table, there was no significant difference in BMI (body mass index), triglyceride, total cholesterol, HDL-cholesterol and LDL-cholesterol levels between the study groups (p>0.05). However, fasting blood glucose levels were significantly increased in patients with positive coronary angiography compared to control subjects (p = 0.009). Our subjects in both control and patient groups were overweight and they had generally high individual variation in the levels of triglyceride producing high standard deviations.

**Table 2:** General characteristics of the study population.

	Positive coronary angiography group (n = 278)	Control group (n = 135)	*p*
Gender (Male/Female) (n)	217/61*	120/15	0.008
Age (year)	58.99±10.56*	48,52±5.87	<0.001
Body Mass Index (kg/m^2^)	28.66±5.14	28.31±4.68	0.668
Triglyceride (mg/dl)	160.93±104.50	165.34±113.10	0.719
Total cholesterol (mg/dl)	189.37±48.23	188.25±35.39	0.846
HDL cholesterol (mg/dl)	41.31±11.41	44.10±13.89	0.139
LDL cholesterol (mg/dl)	131.63±43.06	135.98±36.20	0.308
Fasting blood glucose (mg/dl)	135.52±63.17*	117.70±56.97	0.009

n, number of individuals. Statistical evaluation by Student’s t-test. The results are shown as mean ± Standart Deviation (SD).

*p<0.05. HDL, high density lipoprotein; LDL, low density lipoprotein.

### Relationship between Expression Levels of SIRT1 and eNOS Protein and Levels of TAS, TOS, OSI

SIRT1 protein, TAS, TOS and OSI levels significantly increased in patients as compared to control subjects (p<0.01). In contrast, level of eNOS protein was considerably low in the patients (p = 0.003) (Table 3). Pearson’s correlation test showed a positive correlation between SIRT1 expression with TAS level (p = 0.008) and age (p = 0.014) in patients (Table 4), although there was no significant correlation between SIRT1 activity with eNOS protein, TOS, OSI levels in patients and also in control group (p>0.05).

**Table 3:** Comparison of SIRT1, eNOS protein, TAS, TOS and OSI levels in study population.

	Positive coronary angiography group (n = 278)	Control group (n = 135)	*p*
SIRT1 protein (ng/ml)	2.27±1.25*	1.80±1.01	<0.001
eNOS protein (pg/ml)	867.89±171.32*	922.65±177.62	0.003
TAS (mmolTrolox Equiv./L)	1.58±0.84*	0.96±0.28	<0.001
TOS (µmol H_2_O_2_ Equiv./L)	9.99±13.33*	4.42±9.16	<0.001
OSI (Arbitrary units)	0.83±1.31*	0.47±1.18	0.007

n, number of individuals. Statistical evaluation by the Student’s t-test. The results are shown as mean±Standart Deviation (SD).

*p<0.05.

**Table 4:** Results of Pearson’s correlation between expression level of SIRT1 and other parameters.

	Positive coronary angiography group (n = 278)		Control group (n = 135)	
	r	p	r	p
eNOS protein (pg/ml)	0.089	0.140	0.078	0.372
TAS (mmolTrolox Equiv./L)	0.160	0.008*	0.064	0.458
TOS (µmol H_2_O_2_ Equiv./L)	0.014	0.812	0.049	0.571
OSI	−0.019	0.747	0.055	0.528
Age	0.147	0.014*	0.089	0.303
BMI	−0.049	0.476	−0.159	0.280

n, number of individuals.

*p<0.05.

### Frequencies of SIRT1 (rs7895833 A>G, rs7069102 C>G, rs2273773 C>T) Gene Polymorphisms

The frequencies of genotypes and alleles in *SIRT1* gene in both groups are shown in Table 5. For rs7069102 C>G in intron 4, the frequencies of GG genotype and G allele were significantly higher in patients compared to control group (p<0.001). The risk for cardiovascular disease was increased by 2.4 times in carriers of G allele compared with carriers of C allele (χ^2^: 31.862, p<0.001, OR: 2.431, %95 Cl: 1.779–3.323). In the patients, the rate of having CG genotype and C allele were significantly lower than control subjects (p<0.001) (Table 5).

**Table 5:** Distribution of rs7895833 A>G, rs7069102 C>G and rs2273773 C>T genotypes and alleles in study groups.

	Positive coronary angiography group (n = 278)		Control group (n = 135)		*Statistical results*	
	(%)	(n)	(%)	(n)	?^2^	*p value*
**rs7895833 A>G Genotype**						
AA	72.3	201	72.6	98		
AG	25.5	71	26.7	36	1.123	0.570
GG	2.2	6	0.7	1		
**Allele**						
A	85.1	473	85.9	232	0.106	0.745
G	14.9	83	14.1	38		
**rs7069102 C>G Genotype**						
CC	6.5	18	17.0	23		
CG	32.7*	91	49.6	67	30.215	<0.001
GG	60.8*	169	33.3	45		
**Allele**						
C	22.8*	127	41.9	113	31.862	<0.001
G	77.2*	429	58.1	157		
**rs2273773 C>T Genotype**						
CC	5.0	14	7.4	10		
CT	52.9*	147	78.5	106	32.286	<0.001
TT	42.1*	117	14.1	19		
**Allele**						
C	31.5*	175	46.7	126	18.110	<0.001
T	68.5*	381	53.3	144		

n, number of individuals. Statistical evaluation by the Chi-square test.

*p<0.05.

For rs2273773 C>T in exon 5, the rate of having TT genotype and T allele were significantly increased in patients as compared to control subjects (p<0.001). Correspondingly, the rate of having CT genotype and C allele were significantly lower in the patients compared with controls (p<0.001). The risk for cardiovascular disease was increased by 1.9 times in carriers of T allele compared with carriers of C allele (χ^2^: 18.110, p<0.001, OR: 1.905, %95 Cl: 1.413–2.568). For rs7895833 A>G in the promoter region, there was no significant difference between the groups in the genotypes and allele frequencies (p>0.05).

### Relationship between SIRT1 Gene Polymorphisms and Protein Levels of SIRT1 and eNOS

For rs7895833 A>G, rs7069102 C>G, rs2273773 C>T SNPs, the associations of SIRT1 and eNOS protein levels and distributions of genotypes are shown in Table 6. For rs7895833 A>G, while there was no significant change in the frequency of genotype variations in *SIRT1* polymorphism (Table 5), SIRT1 protein level was significantly increased and eNOS expression was significantly decreased in the patients carrying wild-type (AA) genotype as compared to control (p = 0.009 and p = 0.006, respectively). SIRT1 protein level was also significantly increased in the patients carrying heterozygote mutant (AG) genotype (p = 0.002), whereas eNOS expression was not significantly changed in these patients compared to controls.

**Table 6:** Comparison of rs7895833 A>G, rs7069102 C>G and rs2273773 C>T SNPs with SIRT1 and eNOS protein levels.

	SIRT1 protein (ng/ml)			eNOS protein (pg/ml)		
	Positive coronary angiography group (n = 278)	Control group (n = 135)	*p*	Positive coronaryangiographygroup (n = 278)	Control group(n = 135)	*p*
**rs7895833 A>G Genotype**						
AA	2.23±1.16*	1.86±1.06	0.009	864.20±174.86*	923.57±174.39	0.006
AG	2.39±1.51*	1.64±0.88	0.002	886.78±162.81	918.53±190.75	0.371
GG	2.18±1.07	1.41±0	0.529	767.93±119.39	981.09±0	0.159
**rs7069102 C>G Genotype**						
CC	2.20±1.16	1.61±0.80	0.080	821.52±142.03*	934.35±92.60	0.004
CG	2.22±1.24*	1.82±0.98	0.031	839.23±147.95*	903.52±169.93	0.012
GG	2.31±1.27*	1.87±1.15	0.040	888.26±183.12	945.15±217.56	0.077
**rs2273773 C>T Genotype**						
CC	2.76±1.94	1.79±1.03	0.168	900.16±152.07	945.18±54.81	0.383
CT	2.24±1.18*	1.74±0.88	<0.001	855.12±177.64*	922.60±173.02	0.003
TT	2.25±1.24	2.14±1.54	0.729	880.08±165.22	911.09±241.47	0.481

n: number of individuals. Statistical evaluation by the Student’s t-test. The results are shown as mean±Standart Deviation (SD).

*p<0.05.

For rs7069102 C>G and rs2273773 C>T, both the frequency of genotypes and the level of SIRT1 protein were increased in the patients carrying homozygote mutant genotypes (GG and TT, respectively) (Table 5 and Table 6). However, the increase in the SIRT1 expression in the patients carrying mutant TT genotype for rs2273773 C>T did not reach accepted level of significance. Parallel to this, both the frequency of *SIRT1* genotypes and the level of eNOS protein were significantly decreased in the patients carrying heterozygote genoypes (CG: p = 0.012, and CT: p = 0.003, respectively) for these SNPs. In addition, a significant decrease in the eNOS expression in the patients carrying wild-type (CC: p = 0.004) genotype was noted in a relation with an insignificant decrease in the frequency of this genotype for rs7069102 C>G. On the other hand, a significant increase in the SIRT1 expression in the patients carrying heterozygote mutant (CG: p = 0.031 and CT: p<0.001, respectively) genotypes was noted (Table 6) while a significant decrease in the frequency of *SIRT1* genotypes was observed in these patients for these SNPs (CG: p<0.001 and CT: p<0.001, respectively) (Table 5).

## Discussion

In this study, we analyzed the *SIRT1* gene polymorphisms in patients who had positive coronary angiography and controls in Turkey. Because it may be thought to affect the CVD phenotype by associating body mass index and obesity, glucose tolerance and diabetes, blood pressure, cholesterol metabolism and coronary artery calcification, we performed our experiments on rs7895833 A>G in the promoter, rs7069102 C>G in intron and rs2273773 C>T in exon regions of *SIRT1* gene. The levels of SIRT1 and eNOS expression, TAS, TOS, and OSI were investigated to demonstrate the association between genetic variation and phenotype. In the recent discoveries, the importance of epigenetics in several human diseases were clearly identified. Therefore, by demonstrating the relation between genetic variations and the above parameters, the present study may give a clue for developing essential treatment strategies like life coaching of people about their life styles to decrease the risk of CVD development. Because, the trend of the improvements the quality of care and treatment rather than the prevention of the disease itself becomes important to reduce modifiable risk factors of CVD development.

As mentioned earlier, in the present study, the expression of SIRT1 protein increased in the CVD patients compared to healthy subjects. This is contraversy with some previous studies. Recently, it was found that SIRT1 expression was reduced in patients with CVD [29]. The protection of heart from ROS-mediated cardiotoxicity is lost in a decrease of SIRT1 level [13]. In this study, both our CVD patients (mean age is 58.99±10.56) and control subjects (mean age is 48.52±5.87) are middle-aged. We also found a positive correlation between SIRT1 expression and age. In literature, there are some evidences showing that an increased ROS level due to aging or age-related diseases can directly or indirectly control the activity of SIRT1 [8]. In other words, there is a cross-talk between ROS and SIRT1 protein. It was demonstrated that ROS inhibited JNK phosphatases and stimulated redox-regulated ASK1 kinase, thereby, they activated the JNK1 [30], [31]. Furthermore, JNK1 phosphorylated SIRT1 and this phosphorylation increased the activity of SIRT1 resulting its translocation into the nuclei [32]. Interestingly, this activation specifically deacetylated histone H3. This finding implies that ROS can regulate directly gene expression via the JNK1-SIRT1 link. In a previous transgenic mice study, researchers found that SIRT1 functions in the heart in a dose-dependent manner. For example, moderate elevation of SIRT1 protects cardiomyocytes, and high elevation of SIRT1 decreases cardiac function and causes cardiomyopathy [15]. Furthermore, constitutive overexpression of *SIRT1* gene impairs cardiac function [33]. In another previous study, Sun et al., [34] observed an increase in the expression of SIRT1 protein in the patients with atrial fibrillation. Collectively, they suggested that increased SIRT1 activity may be due to inhibiting the process of oxidative stress. Alcendor et al., [15] found that overexpression of SIRT1 by 2.5 to 7.5 fold decreased age-related cardiac hyperthropy, apoptosis, cardiac dysfunction, and expression of senescence markers while overexpression of SIRT1 by 12.5 fold resulted in oxidative stress, apoptosis, and increased cardiac hyperyhrophy. Increase expression and/or activity of the SIRT1 may be lead to increase histone deacetylation and, thereby, to increase DNA methylation [35]. This SIRT1-mediated epigenetic modification may increase the risk of CVD. These investigations suggest that oxidative stress may cause of pathological increase of SIRT1 expression in CVD patients. In the current study, it was found a positive correlation between SIRT1 activity and TAS level in CVD patients. The increase in the SIRT1 level may also suggest that a compensatory mechanism to increase the antioxidants against oxidative stress in CVD patients. Furthermore, usage of some drugs in the treatment of CVD may also increase the SIRT1 levels [36], [37].

In order to better elucidate the function of SIRT1 protein in the cardiovascular diseases, the other disease marker paramaters like levels of endothelial dysfunction and oxidative stress and their relation with SIRT1 protein level were also investigated. A common feature of many cardiovascular risk factors like hypertension and obesity is caused by endothelial dysfunction which increases atherosclerosis. Endothelial dysfunction could be due to decreased eNOS expression. Endothelial-derived NO protects the cardiovascular system from atherosclerosis by regulating vascular relaxation [38]. Deficiency of eNOS is associated with different risk conditions for cardiovascular disease such as hypertension, ventricular hypertrophy, and diet-induced atherosclerosis [39]–[41]. However, some previous studies suggested that overexpression of eNOS increases the oxidative stress increasing cardiovascular risk factors. In turn, the produced ROS leads to eNOS uncoupling and enzyme dysfunction leading CVD pathophysiology [42]. In our study, the level of eNOS protein was considerably low in the CVD patients whereas SIRT1 level was markedly high as compared to controls.

It was known that oxidative stress generated by ROS may play a crucial role in different types of CVD [43]. Also, a significant relation between TAS levels and extent of CVD has been demonstrated, previously [44]. In our study, we observed a significant increase in the TOS and OSI levels suggesting an increase in the oxidative stress in CVD patients although TAS level also increased. In literature, there are some studies showing an increase in the TOS and OSI and a decrease in the plasma antioxidant capacity in patients with CVD [45]–[47]. In contrast, Aydın et al., [48] found an increase in TAS levels in subjects with coronary atherosclerosis, as in our case. In our study, increase in TAS level in patients could be related with the increase in the SIRT1 protein level because it has a role in the induction of antioxidants [49]. In cardiomyocytes, SIRT1 induces MnSOD and catalase expression and provides resistance of myoblast to oxidative stres due to its antioxidative activity [14], [15].

To understand the relation of CVD phenotype with possible genetic background, three SNPs in *SIRT1* gene were evaluated. The frequencies of mutant GG genotype and mutant G allele for rs7069102 C>G in intron 4 and the frequencies of mutant TT genotype and mutant T allele for rs2273773 C>T in exon 5, were significantly higher in CVD patients as compared to controls. The risk for CVD was increased by 2.4 times in carriers of mutant G allele compared with carriers of wild-type C allele for rs7069102 C>G and 1.9 times in carriers of mutant T allele compared with carriers of wild-type C allele for rs2273773 C>T. According to these results, heterozygote CG genotype for rs7069102 C>G and heterozygote CT genotype for rs2273773 C>T may be protective against to CVD. Therefore, we can say that C allele for both SNPs may be protective allele against CVD development. In our study, we found no association between for rs7895833 A>G SNP and the risk of CVD.

When we considered the relation between SNPs and expression levels of SIRT1 and eNOS, we observed that SIRT1 protein levels for all studied SNPs were significantly increased in the patients carrying heterozygote mutant genotypes as compared to controls. We found that heterozygote CG genotype (rs7069102) and heterozygote CT genotype (rs2273773) may be protective against to CVD. Therefore, the increase in the SIRT1 protein expression may suggest a compensatory mechanism to protect the people from the detrimental effects of CVD. In addition, for rs7069102, the mutant genotype (GG) caused a significant increase in the SIRT1 expression level suggesting that this SNP in the *SIRT1* gene is related with the oxidative stress, thereby, CVD development. Also, increase in SIRT1 protein level of patients carrying wild type genotype of rs7895833 may suggest the deteriorating effects of CVD-induced oxidative stress. An overall decrease in the protein levels of eNOS for all three SNPs was observed in the positive coronary angiography patients, as expected. However, this decrease was significant in patients carrying heterozygote mutant genotypes (CG and CT) for rs7069102 C>G and for rs2273773 C>T and in patients carrying wild-type genotype (AA and CC) for rs7895833 A>G and for rs7069102 C>G. The relation between decreased eNOS expression and CVD risk was also demonstrated previously [50]. However, our result suggested that the decrease in the eNOS levels may not be related with these three *SIRT1* SNPs. This may be a result of the epigenetic modifications of genes. The severity of phenotype of a genetic disease is not related with differences in genotype alone. The individual variation due to epigenetic factors have a great place to explain the observed phenotypes [51]. Further studies are needed to better understand the possibility of pleiotropic effects of *SIRT1* gene variations on CVD.

### Study Limitations

This is an initial Turkish population based study that needs to be larger sample size to better interpret the results for associations between the *SIRT1* polymorphisms and the risk of cardiovascular disease. Another draw-back of the current study is the significantly different ages of control subjects and patients. However, our all subjects are in the range of middle-age and there are similar studies in literature [52].
